# Validation of the Spanish Translation of the Patient Assessment of Chronic Illness Care (PACIC) Survey

**Published:** 2008-09-15

**Authors:** Nirav R Shah, Abraham Aragones, Eric W Schaefer, David Stevens, Marc N Gourevitch, Russell E Glasgow

**Affiliations:** Division of General Internal Medicine, New York University School of Medicine. Dr Shah is also associated with the Center for Health Research, Geisinger Health, Danville, Pennsylvania; Division of General Internal Medicine, New York University School of Medicine, New York, New York, and State University of New York, Downstate Medical Center, Brooklyn, New York; Division of General Internal Medicine, New York University School of Medicine, New York, New York; Division of General Internal Medicine, New York University School of Medicine, New York, New York; Division of General Internal Medicine, New York University School of Medicine, New York, New York; Center for Health Dissemination and Implementation Research, Kaiser Permanente Colorado, Denver, Colorado

## Abstract

**Introduction:**

The Patient Assessment of Chronic Illness Care (PACIC) survey is a patient-centered instrument for evaluating the quality and patient-centeredness of chronic illness care received according to the Chronic Care Model paradigm. This study validates the Spanish translation of the PACIC in an urban, Spanish-speaking population.

**Methods:**

One hundred Spanish-speaking patients with diabetes completed the translated PACIC and sociodemographic and cultural questionnaires. Test-retest reliability was assessed in a subset of 20 patients who completed the questionnaire 2 to 4 months later. Internal consistency was evaluated with Cronbach α. PACIC score and subscale associations with sociodemographic characteristics were examined.

**Results:**

Test-retest reliability for the overall translated PACIC scale was 0.77. Scores were not associated with patient sociodemographic characteristics, including age, country of birth, years living in the United States, or education level (*P* >.05).

**Conclusion:**

The Spanish translation of the PACIC survey demonstrated high reliability, internal consistency, and test-retest reliability. Scores showed no association with sociodemographic or cultural characteristics. The Spanish version can reliably be used to assess care delivered according to the Chronic Care Model in a heterogeneous Spanish-speaking population.

## Introduction

The gap between evidence-based medicine and proactive, patient-centered care has been well documented; the challenge for the near future is to bridge this divide (1-4). A paradigm to achieve this is described by the Chronic Care Model (CCM) (5-7). This model provides a multidimensional framework for improving the quality of chronic care through interventions targeting 6 critical domains: 1) organization of health care, 2) clinical information systems, 3) delivery system design, 4) decision support, 5) self-management support, and 6) community resources. When these CCM goals were applied as part of a Breakthrough collaborative (8), a diverse set of health care teams improved both process and outcome measures for 1 or more chronic illnesses on a panel-wide or population basis (9,10). When applied on a broader scale, the improvements in health care delivery to patients suffering from chronic illness may be further generalizable (1-3,9,10). The urgent need for such change is underscored by the projected increase in people living with a chronic condition in the United States — more than 134 million will be affected by 2020 (11) — and high rates of suboptimal chronic care demonstrated in several studies (11,12).

Evaluating the effect of any complex intervention implemented in a health care delivery system is difficult. Several means are being developed to assess the effect of CCM implementation on systems of care. The Assessment of Chronic Illness Care (ACIC) (13) and the Patient Assessment of Chronic Illness Care (PACIC) (14) survey instruments have been developed to assess CCM implementation at the level of the provider and patient, respectively. The ACIC instrument was developed to measure the extent to which health care teams employ CCM elements with their patients (13); it is completed by care providers. In a pilot trial involving health care teams treating various chronic illnesses, ACIC scores were positively correlated with quality-improvement efforts launched as a component of CCM implementation (13).

Complementing the ACIC is the PACIC, a 20-item patient survey that assesses a patient's receipt of care congruent with key aspects of the CCM for their chronic illness during the previous 6 months (14). This instrument assesses key elements of the CCM: collaborative self-management support and planned, proactive, and population-based care and follow-up. The PACIC provides a patient-centered assessment of the quality of chronic illness care, helping health care teams better understand the level of integration of CCM care in their practices. This tool is the only one available in the literature that measures patients' assessment of chronic disease care received under the CCM. The PACIC avoids the common pitfall of clinician overreporting of the elements of care delivered. As with many patient assessments, the PACIC empowers patients to be evaluators in their health care (15). More importantly, the PACIC may serve as a benchmark of care for health care teams. Changes in health care delivery may be assessed over time by readministering this instrument (14).

The English version of the PACIC questionnaire was developed and validated by Glasgow et al (14). It was developed and tested among 283 patients receiving care for 1 or more chronic conditions under the CCM in a large managed care organization in Washington and Idaho. The PACIC consists of 20 items that were chosen from 46 items designed by national experts on chronic illness care and the CCM. This questionnaire (Appendix I) is divided into 5 subscales to reflect the key components of the CCM: patient activation (3 questions), delivery system design/decision support (3 questions), goal setting/tailoring (5 questions), problem-solving/contextual (4 questions), and follow-up/coordination (5 questions). Each item has a score from 1 (never) to 5 (always). Patients self-report how often they received specific types of medical care during the past 6 months. The questionnaire can be self-administered or staff-administered. The total score for the questionnaire and for each subscale is then tabulated. The concurrent validity of the English version of the PACIC instrument was assessed by correlating its scores with results from other instruments that measure delivery of primary care (14). The authors found good reliability and good face, construct, and concurrent validity.

The PACIC was initially tested and validated in a population of mostly white, English-speaking patients with various chronic illnesses (14), and more recently with a sample of English-speaking patients with diabetes (16). With the well-documented challenges of health disparities in the United States (17) and the rapid growth of the Hispanic/Latino population in the United States (18), there is now a need to assess chronic care services received by this group. The aim of our study was to test and validate the psychometric properties of the Spanish translation of the PACIC and to better understand the effect of the CCM in this population.

## Methods

### PACIC questionnaire translation

Using accepted guidelines for translation–back translation (19-21), the English version of the PACIC questionnaire was translated into Spanish by 2 native Spanish speakers fluent in English and medical terminology. Two different translators then independently back translated the Spanish version into English, with any differences resolved by consensus. The back-translated English version was then compared with the original English version to ensure that no loss of meaning or context occurred during the translation process. The translated questionnaire is in Appendix II.

### Population

For this cross-sectional validation study, we recruited Spanish-speaking Hispanic patients with diabetes who were receiving care at an adult ambulatory care clinic of a large municipal hospital in New York City. This clinic was selected because the physicians and staff have participated in a Breakthrough Series Collaborative led by Ed Wagner of the MacColl Institute, a leading proponent of the CCM. Components of the CCM have been integrated into aspects of care in this clinic since 2004, and biannual feedback is discussed at a citywide CCM collaborative.

A trained bilingual research assistant approached all patients (previously identified as having type 2 diabetes) in the waiting room of the clinic before their visit with their provider. Those who acknowledged speaking Spanish, met all the inclusion criteria, and consented to participate in the study were administered the translated Spanish PACIC questionnaire and were asked additional demographic and cultural questions. Clinical information was obtained by an electronic chart review. All patients recruited in the study self-reported Spanish as their primary language, were aged 18 years or older, reported using the clinic as their primary source of medical care, and had at least 1 visit in the 6 months before the enrollment visit. To reduce variability in reporting health care received for chronic illnesses, we selected patients who had a chronic medical condition in common: type 2 diabetes. Of the 120 patients who were approached and met criteria to participate in the study, 20 refused to participate, most often because of time constraints (80% of refusals).

### Analyses

We computed means, standard deviations, confidence intervals, and distributions of scores of the overall PACIC and its 5 subscales. All calculations were conducted by using SPSS 14.0 for Windows (SPSS Inc, Chicago, Illinois). Data collected on demographic factors included age, sex, marital status, educational level, insurance status, country of birth, the number of years living in the United States, and number of chronic conditions the patient had. From 2 to 4 months after the initial survey, 20 patients (20% of the overall sample) were randomly selected by using SPSS's simple random sampling method. These patients completed a telephone interview with the translated Spanish PACIC. We evaluated reliability and internal consistency of the PACIC and its subscales using Cronbach α (22,23). We correlated patient demographics and PACIC scores by using Spearman rank order correlation because of the nonnormal distribution of the PACIC scores. We performed factor loading analysis to confirm the correlation of the independent items within each subscale by using the varimax rotation (22,24), a criterion that maximizes the variance of the squared elements in the factor matrix. We selected this method because we had no reason to suspect a principal factor and because interpretation would be easier.

This study was approved by the New York University School of Medicine institutional review board.

## Results

### Respondent characteristics

The 100 Spanish-speaking patients with diabetes completed the translated PACIC between April 2006 and February 2007. Seventy-nine percent of participants were women, 46% had an education level below sixth grade, and 10% were uninsured. Most patients (69%) were born in Spanish-speaking Caribbean countries (Table 1). No demographic characteristics, including number of years in the United States, were significantly associated with overall PACIC scores or subscales or had correlations higher than 0.18 (Table 2). We found no significant differences in patient demographic characteristics (age, sex, and number of years since moving to the United States) between those who consented to participate in the study and those who did not.

### PACIC reliability

The average overall PACIC score was 3.17 (SD, 0.82). Mean subscale scores ranged from 2.50 to 3.95 (Table 3). Neither ceiling nor floor effects were observed (ie, scores were not clustered near the top or bottom range of the scale), suggesting good discrimination potential. Internal consistency of the overall PACIC (Cronbach α) was 0.87 and test-retest reliability (Cronbach α) was 0.77 (Table 3), suggesting excellent internal consistency and test-retest reliability. For internal consistency, all subscales had a Cronbach α greater than 0.6 for the complete sample, which is acceptable for brief scales (23,25). One subscale, delivery system design/decision support, had Cronbach α <0.6 in the test-retest component.

### Confirmatory factor analysis

The results of the factor analysis for each of the PACIC subscales are listed in Table 4. Most of the items correlated highly on the proposed scales. Of the 20 items, 13 had factor loading (α) greater than 0.7 and only 1 item had factor loading less than 0.6 (question 17, α = 0.48).

## Discussion

The Spanish translation of the PACIC questionnaire demonstrated high reliability, internal consistency, and test-retest reliability, extending the applicability of this instrument to Spanish-speaking patients. Given the increase in the proportion of Spanish-speaking patients in the United States (18) and the increased use of the CCM to guide system change in hospital and community settings (26,27), there is a pressing need for practical, validated tools to evaluate the CCM among Spanish-speaking persons. We believe this translated PACIC questionnaire begins to fill that void.

PACIC scores should not be related to patients' demographic characteristics, as noted by the developers of the original English version of this scale (16). Indeed, we found that PACIC scores in the Spanish-speaking population studied were not correlated with the number of chronic medical conditions or with sociodemographic characteristics such as age, number of years in the United States, level of education, or country of origin. This suggests that overall, the translation was successful despite possible variations in other unmeasured factors such as cultural aspects and health literacy.

The factor analysis demonstrated that almost every question fit well into its particular subscale. The exception was question 17, but the difference did not reach statistical significance. This question asks whether patients were encouraged to attend community programs to aid in their chronic illness care. Patients possibly understood the medical clinic itself to be their "community" health care resource, and they therefore did not pursue other community resources.

The population studied was limited to patients with diabetes to reduce the variability in care received, and our study provides a direct cross-validation of the Spanish version with the English-speaking diabetes validation (16). Although patients with other chronic medical conditions may view their medical care differently from those with diabetes, the original English PACIC was tested in populations with various chronic conditions yet displayed no differences in its psychometric properties across these conditions (14).

This study recruited patients from only 1 health care setting. Conceivably, the 6 domains of the CCM might translate differently depending on the environment. For example, patients might view their care differently in a specialty clinic relative to a primary care setting. Though this may affect the generalizability of the results of our assessment of the Spanish translation of the PACIC questionnaire, its essential reliability and consistency, demonstrated herein, should not be affected. We encourage other researchers to replicate this study among Spanish-speaking patients receiving care under the CCM for different chronic conditions.

Glasgow et al found that the PACIC was useful in assessing care delivered to patients with diabetes and encouraged its integration into quality improvement initiatives (16). Our results confirm that the Spanish translation of the PACIC questionnaire can be used in a mixed Hispanic population and still retain excellent psychometric properties despite the potential for cultural or ethnic variations. Once additional validation studies are conducted on different, independent samples, we believe this tool can be used to assess the implementation of the CCM in various clinical settings with Hispanic populations and to aid in both formal evaluation and quality improvement projects to enhance the delivery of patient-centered health care among Spanish-speaking populations.

## Figures and Tables

**Table 1:** Sociodemographic Characteristics of Spanish-Speaking Patients With Diabetes, Spanish Translation of the Patient Assessment of Chronic Illness Care Survey (N = 100), New York City, 2006–2007

**Characteristic**	**Value**
**Age, y**	
Mean (SD)	63.7 (10.7)
Range	37-80
**Sex, %**	
Male	21
Female	79
**Marital status, %**	
Married	33
Widowed	28
Single	16
Divorced	11
Other	12
**Education level, %**	
≤6th grade	46
7th-12th grade	43
More than high school	11
**Insurance status, %**	
Insured	90
Uninsured	10
**Country of birth, %**	
Puerto Rico	36
Dominican Republic	33
Mexico	8
United States	5
Other	18
**No. of years living in United States**	
Mean (SD)	34.9 (16.4)
Range	1-60
**No. of chronic conditions (excluding diabetes)**	
Mean (SD)	4.2 (1.7)
Range	0-9
**High blood pressure, %**	76
**Pain, %**	73
**Arthritis, %**	60
**Depression, %**	32
**Asthma, %**	21

**Table 2:** Correlation^a^ of Demographic Characteristics of Spanish-Speaking Patients With Diabetes and Their Scores From a Spanish Translation of the Patient Assessment of Chronic Illness Care (PACIC) Survey (N = 100), New York City, 2006–2007

**Scale**	Age	% Male	Education Level	Insurance Status	No. of Years Living in United States	No. of Chronic Conditions (Excluding Diabetes)
**Overall PACIC**	0.02	−0.01	0.02	0.14	0.01	0.03
Patient activation	−0.17	0.03	0.14	−0.09	0.03	0.02
Delivery system design/decision support	0.09	−0.01	−0.004	0.11	0.002	0.04
Goal setting/tailoring	0.09	0.05	−0.08	0.18	0.09	0.08
Problem solving/contextual	0.04	−0.11	−0.05	0.17	−0.01	−0.05
Follow-up/coordination	0.02	−0.02	0.14	0.12	−0.04	0.05

a Spearman rank order correlation. No values are statistically significant (*P* >.05 for all).

**Table 3:** Scores and Reliability of a Spanish Translation of the Patient Assessment of Chronic Illness Care (PACIC) Survey Among Spanish-Speaking Patients With Diabetes (N = 100), New York City, 2006–2007

Scale	Mean (SD)	Cronbach α	

		Internal Consistency (N = 100)	Test-Retest (n = 20)
**Overall PACIC**	3.17 (0.82)	0.87	0.77
Patient activation	2.93 (1.25)	0.61	0.61
Delivery system design/decision support	3.95 (0.98)	0.60	0.50
Goal setting/tailoring	3.09 (1.08)	0.73	0.69
Problem solving/contextual	3.75 (1.10)	0.70	0.62
Follow-up/coordination	2.50 (0.90)	0.60	0.74

**Table 4:** Instrument Properties and Confirmatory Analysis of a Spanish Translation of the Patient Assessment of Chronic Illness Care Survey Scale Among Spanish-Speaking Patients With Diabetes (N = 100), New York City, 2006–2007

Scale/Question^a^	Eigenvalues			Factor Loading (α)

	Total	% of Variance	Cumulative %	
**Patient activation**				
1	1.70	56.73	56.73	0.77
2	0.81	27.07	83.79	0.84
3	0.49	16.21	100.00	0.63
**Delivery system design/decision support**				
4	1.71	57.11	57.11	0.74
5	0.76	25.44	82.55	0.69
6	0.52	17.45	100.00	0.82
**Goal setting/tailoring**				
7	2.44	48.87	48.87	0.78
8	0.77	15.49	64.35	0.76
9	0.73	14.63	78.98	0.65
10	0.65	13.02	92.00	0.61
11	0.40	8.00	100.00	0.67
**Problem solving/contextual**				
12	2.16	53.91	53.91	0.72
13	0.95	23.65	77.57	0.80
14	0.53	13.31	90.87	0.68
15	0.37	9.13	100.00	0.73
**Follow-up/coordination**				
16	1.97	39.34	39.34	0.72
17	1.06	21.11	60.45	0.48
18	0.93	18.66	79.11	0.86
19	0.60	11.97	91.07	0.81
20	0.45	8.93	100.00	0.80

a The questions are listed in Appendix I (English) and Appendix II (Spanish).

## Appendices

### Appendix I. English Version of the Patient Assessment of Chronic Illness Care Questionnaire

Source: Reference 14.

**Table:**

**Assessment of Care for Chronic Conditions**					
Staying healthy can be difficult when you have a chronic condition. We would like to learn about the type of help with your condition you get from your health care team. This might include your regular doctor, his or her nurse, or physician's assistant who treats your illness. Your answers will be kept confidential and will not be shared with your physician or clinic.					
Over the past 6 months, when I received care for my chronic conditions, I was:					

	None of the Time	A Little of the Time	Some of the Time	Most of the Time	Always
1. Asked for my ideas when we made a treatment plan.	q _1_	q _2_	q _3_	q _4_	q _5_
2. Given choices about treatment to think about.	q _1_	q _2_	q _3_	q _4_	q _5_
3. Asked to talk about any problems with my medicines or their effects.	q _1_	q _2_	q _3_	q _4_	q _5_
4. Given a written list of things I should do to improve my health.	q _1_	q _2_	q _3_	q _4_	q _5_
5. Satisfied that my care was well organized.	q _1_	q _2_	q _3_	q _4_	q _5_
6. Shown how what I did to take care of myself influenced my condition.	q _1_	q _2_	q _3_	q _4_	q _5_
7. Asked to talk about my goals in caring for my condition.	q _1_	q _2_	q _3_	q _4_	q _5_
8. Helped to set specific goals to improve my eating or exercise.	q _1_	q _2_	q _3_	q _4_	q _5_
9. Given a copy of my treatment plan.	q _1_	q _2_	q _3_	q _4_	q _5_
10. Encouraged to go to a specific group or class to help me cope with my chronic condition.	q _1_	q _2_	q _3_	q _4_	q _5_
11. Asked questions, either directly or on a survey, about my health habits.	q _1_	q _2_	q _3_	q _4_	q _5_
12. Sure that my doctor or nurse thought about my values, beliefs, and traditions when they recommended treatments to me.	q _1_	q _2_	q _3_	q _4_	q _5_
13. Helped to make a treatment plan that I could carry out in my daily life.	q _1_	q _2_	q _3_	q _4_	q _5_
14. Helped to plan ahead so I could take care of my condition even in hard times.	q _1_	q _2_	q _3_	q _4_	q _5_
15. Asked how my chronic condition affects my life.	q _1_	q _2_	q _3_	q _4_	q _5_
16. Contacted after a visit to see how things were going.	q _1_	q _2_	q _3_	q _4_	q _5_
17. Encouraged to attend programs in the community that could help me.	q _1_	q _2_	q _3_	q _4_	q _5_
18. Referred to a dietitian, health educator, or counselor.	q _1_	q _2_	q _3_	q _4_	q _5_
19. Told how my visits with other types of doctors, like an eye doctor or surgeon, helped my treatment.	q _1_	q _2_	q _3_	q _4_	q _5_
20. Asked how my visits with other doctors were going.	q _1_	q _2_	q _3_	q _4_	q _5_

### Appendix II. Spanish Version of the Patient Assessment of Chronic Illness Care Questionnaire

Source for original English version: Reference 14.

**Table:**

**Evaluación del Cuidado de Enfermedades/Condiciones Crónicas**					
Mantenerse saludable puede ser difícil cuando tiene una enfermedad crónica. Quisiéramos saber el tipo de ayuda que usted recibe de su equipo de cuidado de la salud. Esto podría incluir aquellos que tratan su enfermedad como su doctor regular, su enfermero/a o asistente medico. Sus respuestas se mantendrán confidenciales y no serán compartidas con su medico o la clínica.					
En los últimos 6 meses, cuando recibí cuidado medico por mi enfermedad crónica:					

	Nunca	Pocas Veces	Algunas Veces	La Mayoría del Tiempo	Siempre
1. Me preguntaron lo que yo pensaba cuando tomamos la decisión del plan para mi tratamiento.	q _1_	q _2_	q _3_	q _4_	q _5_
2. Me dieron diferentes alternativas de tratamientos para que lo pensara.	q _1_	q _2_	q _3_	q _4_	q _5_
3. Me preguntaron si tengo algún problema con mis medicamentos o sus efectos.	q _1_	q _2_	q _3_	q _4_	q _5_
4. Me dieron una lista escrita de cosas que debo hacer para mejorar mi salud.	q _1_	q _2_	q _3_	q _4_	q _5_
5. Estaba satisfecho que mi cuidado medico estaba bien organizado.	q _1_	q _2_	q _3_	q _4_	q _5_
6. Me mostraron como lo que yo he echo para cuidarme influencio mi enfermedad/condición.	q _1_	q _2_	q _3_	q _4_	q _5_
7. Me pidieron que hable sobre mis metas en cuanto al cuidado de mi enfermedad/condición.	q _1_	q _2_	q _3_	q _4_	q _5_
8. Me ayudaron a establecer metas específicas para mejorar mi alimentación y ejercicios.	q _1_	q _2_	q _3_	q _4_	q _5_
9. Me dieron una copia del plan para mi tratamiento.	q _1_	q _2_	q _3_	q _4_	q _5_
10. Me animaron a que vaya a un grupo específico o a una clase para que me ayude a hacer frente a mi enfermedad/condición crónica.	q _1_	q _2_	q _3_	q _4_	q _5_
11. Me hicieron preguntas, directamente o a través de un cuestionario, acerca de mis hábitos relacionados a la salud.	q _1_	q _2_	q _3_	q _4_	q _5_
12. Estaba seguro que mi medico o enfermero/a pensó en mis valores, creencias y tradiciones cuando me recomendaron el tratamiento.	q _1_	q _2_	q _3_	q _4_	q _5_
13. Me ayudaron a crear un plan para mi tratamiento que yo pudiera realizar en mi vida cotidiana.	q _1_	q _2_	q _3_	q _4_	q _5_
14. Me ayudaron a planificar con tiempo para poder cuidar de mi enfermedad/condición aun en tiempos difíciles.	q _1_	q _2_	q _3_	q _4_	q _5_
15. Me preguntaron como mi condición/enfermedad crónica afecta mi vida.	q _1_	q _2_	q _3_	q _4_	q _5_
16. Me contactaron después de mi visita para ver como iban las cosas.	q _1_	q _2_	q _3_	q _4_	q _5_
17. Me animaron a que vaya a programas en mi comunidad que me podrían ayudar.	q _1_	q _2_	q _3_	q _4_	q _5_
18. Me enviaron a un dietista/nutricionista, educador de la salud o a un consejero.	q _1_	q _2_	q _3_	q _4_	q _5_
19. Me dijeron como las visitas a otros médicos, como el oftalmólogo o el cirujano ayudaron con mi tratamiento.	q _1_	q _2_	q _3_	q _4_	q _5_
20. Me preguntaron como estaban yendo las visitas a los otros médicos.	q _1_	q _2_	q _3_	q _4_	q _5_
